# Supplementary material for: Factors that motivate men who have sex with men in Berlin, Germany, to use or consider using HIV pre-exposure prophylaxis—A multi-methods analysis of data from a multicentre survey
Source: PLoS One. 2021 Nov 18;16(11):e0260168. doi: 10.1371/journal.pone.0260168 (PMC8601504; doi:10.1371/journal.pone.0260168)
Supplement: S1 Table — (DOCX) [file pone.0260168.s001.docx]

**S1 Table:** **Demographic data and sexual risk behaviour of participants in analysis sample and participants who were neutral about or potentially interested in taking PrEP but who did not answer the question about their main motivation**

|  | | **Analysis sample** | | **No motivation reported and did not disagree or strongly disagree with statement**  **“I would like to take PrEP”** | |  |
| --- | --- | --- | --- | --- | --- | --- |
|  | | N = 228 | | N = 104 | |  |
| Age | | | | | | |
|  | Mean (SD) | 36.4 (10.8) |  | 36.7 (11.9) |  |  |
|  | Min-Max | 20-79 |  | 19-73 |  |  |
|  | Not stated | 9 |  | 0 |  |  |
| Highest degree or level of school (N, %) | | | | | | |
|  | Primary education | 0 | (0.0%) | 0 | (0.0%) |  |
|  | Secondary education up to year 10* | 21 | (9.2%) | 12 | (11.5%) |  |
|  | Secondary education with apprenticeship | 11 | (4.8%) | 5 | (4.8%) |  |
|  | Secondary education up to year 12** | 41 | (18.0%) | 17 | (16.4%) |  |
|  | University degree | 151 | (66.2%) | 70 | (67.3%) |  |
|  | Not stated | 4 | (1.8%) | 0 | (0.0%) |  |
| Financial situation (N, %) | | | | | | |
|  | Not always have enough money | 19 | (8.3%) | 19 | (18.3%) |  |
|  | Enough money | 104 | (45.6%) | 43 | (41.4%) |  |
|  | More than enough money | 102 | (44.7%) | 41 | (39.4%) |  |
|  | Not stated | 3 | (1.3%) | 1 | (1.0%) |  |
| Place of residence (N, %) | | | | | | |
|  | Berlin | 213 | (93.4%) | 100 | (96.2%) |  |
|  | Other city in Germany | 7 | (3.1%) | 2 | (1.9%) |  |
|  | Small town / rural area in Germany | 0 | (0.0%) | 0 | (0.0%) |  |
|  | Other country | 5 | (2.2%) | 2 | (1.9%) |  |
|  | Not stated | 3 | (1.3%) | 0 | (0.0%) |  |
| Family origins (N, %) | | | | | | |
|  | Participant & both parents born in Germany | 132 | (57.9%) | 59 | (56.7%) |  |
|  | One parent born outside Germany | 17 | (7.5%) | 6 | (5.8%) |  |
|  | Both parents born outside Germany | 19 | (8.3%) | 7 | (6.7%) |  |
|  | Participant born outside Germany | 56 | (24.6%) | 32 | (30.8%) |  |
|  | Not stated | 4 | (1.8%) | 0 | (0.0%) |  |
| Current HIV status (N, %) | | | | | | |
|  | HIV negative | 198 | (86.8%) | 85 | (81.7%) |  |
|  | Not sure | 24 | (10.5%) | 17 | (16.4%) |  |
|  | Not stated | 6 | (2.6%) | 2 | (1.9%) |  |
| Present or past use of PrEP | | | | | | |
|  | No | 163 | (71.5%) | 88 | (84.6%) |  |
|  | Yes | 65 | (28.5%) | 14 | (13.5%) |  |
|  | Not stated | 0 | (0.0%) | 2 | (1.9%) |  |
| STI diagnosis in the past six months (N, %) | | | | | | |
|  | No | 168 | (73.7%) | 90 | (86.5%) |  |
|  | Yes | 57 | (25.0%) | 13 | (12.5%) |  |
|  | Not stated | 3 | (1.3%) | 1 | (1.0%) |  |
| Role when having anal sex (N, %) | | | | | | |
|  | No anal sex | 7 | (3.1%) | 6 | (5.8%) |  |
|  | Bottom only | 21 | (9.2%) | 4 | (3.9%) |  |
|  | More bottom than top | 60 | (26.3%) | 13 | (12.5%) |  |
|  | Top and bottom (versatile) | 58 | (25.4%) | 41 | (39.4%) |  |
|  | More top than bottom | 49 | (21.5%) | 23 | (22.1%) |  |
|  | Top only | 30 | (13.2%) | 14 | (13.5%) |  |
|  | Not stated | 3 | (1.3%) | 3 | (2.9%) |  |
| Number of anal sex partners in the past six months (N, %) | | | | | | |
|  | None | 14 | (6.1%) | 16 | (15.4%) |  |
|  | 1 | 22 | (9.6%) | 25 | (24.0%) |  |
|  | 2 to 5 | 67 | (29.4%) | 29 | (27.9%) |  |
|  | 6 to 10 | 44 | (19.3%) | 18 | (17.3%) |  |
|  | More than 10 | 76 | (33.3%) | 14 | (13.5%) |  |
|  | Not stated | 5 | (2.2%) | 2 | (1.9%) |  |
| Number of anal sex partners without using condom in the past six months (N, %) | | | | | | |
|  | None | 62 | (27.2%) | 43 | (41.4%) |  |
|  | 1 | 54 | (23.7%) | 32 | (30.8%) |  |
|  | 2 to 5 | 71 | (31.1%) | 25 | (24.0%) |  |
|  | 6 to 10 | 21 | (9.2%) | 1 | (1.0%) |  |
|  | More than 10 | 17 | (7.5%) | 2 | (1.9%) |  |
|  | Not stated | 3 | (1.3%) | 1 | (1.0%) |  |
