# Supplementary material for: Factors that motivate men who have sex with men in Berlin, Germany, to use or consider using HIV pre-exposure prophylaxis—A multi-methods analysis of data from a multicentre survey
Source: PLoS One. 2021 Nov 18;16(11):e0260168. doi: 10.1371/journal.pone.0260168 (PMC8601504; doi:10.1371/journal.pone.0260168)
Supplement: Attachment — Submitted filename: Response to Reviewers.docx [file pone.0260168.s003.docx]

**Response to the academic editor’s and reviewers’ comments**

**Manuscript:** Factors that motivate men who have sex with men in Berlin, Germany, to use or consider using HIV pre-exposure prophylaxis – A multi-methods analysis of data from a multicentre survey [PONE-D-21-17716]

> We would like to thank the academic editor and each of the reviewers for their careful review of our manuscript and their thorough and very helpful suggestions for improving it. We feel that the manuscript has benefitted greatly as a result. Please find our replies to each of their points below, including details on how we have implemented their suggestions. Please note that all line numbers below refer to the file ‘Revised Manuscript with Track Changes’. (If the reviewers prefer that the line numbers refer to the clean file without track changes, we would be happy to send a revised point-by-point reply letter with these instead.)

**RESPONSES TO ACADEMIC EDITOR’S COMMENTS:**

**Academic editor comments**

***Comment 1:*** *Thank you for submitting your manuscript to PLOS ONE. After careful consideration, we feel that it has merit but does not fully meet PLOS ONE’s publication criteria as it currently stands. Therefore, we invite you to submit a revised version of the manuscript that addresses the points raised during the review process. Both reviewers found the paper interesting and valuable, but they raised a number of important issues that need to be addressed. These include clarifying the study background and reframing the rationale; referencing and addressing a number of relevant studies that are omitted (in Intro and Discussion); more fully describing the methods and data analysis; and providing context on PrEP availability in Germany and the broader EU context for international readers.*

**Reply 1:** We are very grateful for the comprehensive, critical and constructive review our paper has received from the reviewers and are also thankful for the opportunity to resubmit the paper for your consideration. We provide details below on how we have implemented each of their suggestions and have addressed the important issues summarised in your comment above.

***Comment 2:*** *Please ensure that your manuscript meets PLOS ONE's style requirements, including those for file naming. The PLOS ONE style templates can be found at*

*https://journals.plos.org/plosone/s/file?id=wjVg/PLOSOne_formatting_sample_main_body.pdf and*

[*https://journals.plos.org/plosone/s/file?id=ba62/PLOSOne_formatting_sample_title_authors_affiliations.pdf*](https://journals.plos.org/plosone/s/file?id=ba62/PLOSOne_formatting_sample_title_authors_affiliations.pdf)

**Reply 2:** We have reviewed PLOS ONE’s style requirements again and have now ensured that the manuscript and file names meet these in full.

***Comment 3:*** *Please include additional information regarding the survey or questionnaire used in the study and ensure that you have provided sufficient details that others could replicate the analyses. For instance, if you developed the survey or questionnaire as part of this study and it is not under a copyright more restrictive than CC-BY, please include a copy, in both the original language and English, as Supporting Information. If the questionnaire is published, please provide a citation to the (1) questionnaire and/or (2) original publication associated with the questionnaire.*

**Reply 3:** In the Methods section, we include the sentence about the availability of our survey questionnaire and the related quantitative publication, both of which are freely available from PLOS ONE:

‘The questionnaires and information cover sheet can be accessed in full, in both English and German, in the publication of the quantitative study results by Werner et al. (2018) [30].’ (ll. 262-264)

(30. Werner RN, Gaskins M, Ahrens J, Jessen H, Kutscha F, Mosdzen R, et al. Knowledge and use of HIV pre-exposure prophylaxis among men who have sex with men in Berlin – A multicentre, cross-sectional survey. PLOS ONE. 2018;13(9):e0204067.)

If more information is needed or if we need to upload a PDF of the survey questionnaire separately for the current publication, we are very happy to do so.

**Specific Responses:**

**RESPONSES TO REVIEWER #1:**

***GENERAL***

***Comment 1****: Thank you for the opportunity to review “Factors that motivate men who have sex with men in Berlin, Germany, to use or consider using HIV pre-exposure prophylaxis – A mixed-methods analysis of data from a multi-centre survey”. The authors conducted a thorough analysis of both quantitative and qualitative data from a cross-sectional survey conducted with a relatively large sample of MSM with complete data on the qualitative component (n=228). The manuscript contributes to the broader literature about the importance of understanding social and structural factors in PrEP uptake. The authors’ conclusion that health professionals seeking to increase PrEP uptake need take a holistic approach that emphasizes multiple motivations is well-founded in their data. My enthusiasm for this manuscript is tempered by three major concerns. Specifically, the manuscript is in need of: 1) a more thorough literature review that draws on existing research about motivations for PrEP use; 2) clarification of the mixed-methods methodology; and 3) re-structuring of the methods/results. In addition to providing more details regarding these concerns, I offer some additional feedback by manuscript section below for the authors’ consideration.*

**Reply 1:** We are very grateful for the reviewer’s thorough, critical and constructive comments and suggestions, and feel that the paper has benefitted substantially from our having implemented them. We provide specific details below on how we have done so, but we feel it might be helpful to outline these broadly here:

1) In the Introduction and Discussion sections, we now include a much more thorough treatment of existing qualitative and quantitative research about motivations for PrEP use. This comprises all of the literature suggested by the reviewer, as well as a range of other pertinent studies. In doing so, we hope we now both better recognise the work of previous researchers and reflect upon how our own findings are related to their results. This has also helped us provide a more sophisticated framing of the rationale for our study and how our findings fit into the broader evidence base. Lastly, the more thorough literature review has allowed us to expand on our findings about the need for a more holistic approach to PrEP implementation.
2) We have clarified our methodology and specify, as the reviewer has suggested, that ours is a multi-methods rather than a mixed-methods approach.
3) We have revised and restructured most of the Methods section in accordance with the reviewer’s suggestions. In particular, we explain in greater detail how we developed our coding framework and how we applied it to analyse the qualitative data from our survey.

***ABSTRACT***

***Comment 2****: I’m not completely clear based on the results presented in the abstract how recognition of multiple and varied motivations may reassure practitioners concerned about condom use in relation to PrEP, particularly when the authors note that almost 1/5 intended not to use condoms. I would consider removing that line, as it requires too much context to be flushed out in an abstract in a nuanced way, but is explored in more depth in the discussion section of the manuscript.*

**Reply 2:** We agree and have removed this line from the abstract. The more in-depth treatment of the subject has been left in the discussion section of the manuscript, as suggested. (Change made to ll. 73-74)

***Comment 3****: I’m unclear exactly what the authors mean by ‘categories’ in the sentence “…responses overall were heterogeneous, even within categories”; additional clarification would be most helpful.*

**Reply 3:** Here (and in a similar sentence in the conclusion section) we are referring to a passage in our Discussion in which we highlight the heterogeneity of responses both overall and even within individual categories that would seem, at first glance, to be one-dimensional, such as ‘safety and protection’:

‘Although these multiple motivations often fell into the same category (most frequently into that of safety and protection), it is important to bear in mind that even in a category as seemingly one-dimensional as “safety and protection”, a broad range of subcategories could be identified, including protection during periods of anticipated increased risk, self-perceptions of being at high risk, and additional protection against HIV while using a condom.’ (ll. 624-630)

We agree with the reviewer, however, that the wording in the abstract and conclusion is not clear enough, and there is not enough space to flesh this out properly. To address this, we have removed the phrase ‘even within categories’ from these two sections while leaving the more in-depth discussion in the discussion section. (Changes made to l. 76 and ll. 792-793)

***Comment 4****: I appreciate the last line of the conclusion as a clear message for practitioners that is well-founded in the data.*

**Reply 4:** Thank you very much.

***INTRODUCTION***

***Comment 5****: The authors’ strong opening paragraphs which detail the background of PrEP approval, the importance of PrEP to reducing HIV disparities particularly among MSM, and context of PrEP uptake in Berlin, Germany. In paragraph 2, it would be helpful for the authors to hypothesize or provide data pertaining to why those who were likely to use PrEP were not able to access it.*

**Reply 5:** Thank for the positive assessment of our opening paragraphs, and for this suggestion, which we have implemented by adding the following sentence to the end of the paragraph in question and removing a similar sentence from the limitations section of the Discussion:

‘The most frequently reported barriers to PrEP use by these participants were worries about adverse effects, cost, not having a doctor who prescribes it, and, despite a high awareness of PrEP, a lack of information on its pros, cons and proper use [30].’ (Changes made to ll. 116-119, ll. 772-775)

***Comment 6:*** *I would also be interested to know a bit more about why policy makers in many countries are debating integration of PrEP into publicly-funded systems, but recognize that this may be beyond the scope of the paper.*

**Reply 6:** We agree that this is an interesting and important point. In particular, we were thinking of the situation in parts of Central, Eastern and Southeastern Europe, where PrEP is not always readily available through public or even private providers, and its costs, depending on the country or region, are not always covered by public health insurance or public health systems. Sometimes the health policy decision-making has been accompanied by strong debate and even homophobic media coverage (the controversy surrounding the early funding decisions and press releases of the commissioning body called NHS England being one good case in point). Because this is indeed an important subject for people to be aware of, we have added several references that describe the current piecemeal situation in Europe (including the only very recent approval of PrEP coverage through the English NHS, unlike in Scotland, where PrEP has been available through local sexual health clinics since mid-2017). (Changes made to l. 106). We have otherwise not gone into greater detail in the main body of the text because we feel it would go beyond the scope of this paper.

***Comment 7:*** *In between the lines starting “As of late 2019…” and “This is unfortunate…” it would be helpful to add one line and associated references that refers to the disproportionate prevalence of HIV among MSM in the US context (see: https://www.cdc.gov/hiv/group/msm/index.html).*

**Reply 7:** This is a crucial point to make, and we have now added this information for both the US and Europe, albeit with data on current incidence rather than prevalence. (We can add the latter if the reviewer thinks this would be advisable; however, we are conscious of the length of the paper as it currently stands.) Please note that we have added this information to the top of the paragraph, a bit earlier than the reviewer suggests, after some consideration back and forth as to which location might have the best flow. The relevant sentence is as follows:

‘Men who have sex with men (MSM) continue to be the population most affected by HIV in the United States (US) and Europe, comprising 69% of new HIV diagnoses in the US [1] and 52% of new diagnoses (where the route of transmission was known) in the European Union (EU)/European Economic Area in 2018 [2].’ (ll. 83-87)

***Comment 8:*** *In terms of flow, I would consider switching the order of paragraphs 3 on the PrEP continuum of care and paragraph 4 on barriers to PrEP uptake.*

**Reply 8:** We understand entirely why the reviewer has suggested this, but after trying it both ways a number of times, we feel it would be best to start with the PrEP continuum of care paragraph for the following two reasons: a) The paragraph ends by describing how each step or stage in the continuum has its own set of barriers and facilitators, and this flows naturally into a paragraph on these; b) As the reviewer will see, we have extensively revised the paragraph on the PrEP continuum to cover the various different models and the extensive previous work done in this area, following his/her very helpful advice. (ll. 121-151) By keeping the PrEP continuum paragraph in its current position, we are now able to introduce the subject of the predominantly biomedical approach to HIV prevention early in the manuscript where it can have a more powerful rhetorical effect. We are nevertheless still amendable to reversing the order of the paragraphs if the reviewer feels it is necessary.

***Comment 9****: I appreciate the authors’ attentiveness to social and structural barriers to PrEP uptake and considerations of a PrEP continuum of care. To that end, there is some literature that should be integrated into the PrEP continuum of care paragraph as well as used to contextualize the discussion. Please see:*

*https://www.ncbi.nlm.nih.gov/pmc/articles/PMC6253066/*

*https://www.ncbi.nlm.nih.gov/pmc/articles/PMC5315535/*

*https://journals.lww.com/jaids/Abstract/2021/04150/The_PrEP_Cascade_in_a_National_Cohort_of.4.aspx?context=LatestArticles*

*While the latter two articles follow more biomedically-oriented conceptualizations of a PrEP cascade, the first article (Newman et al., 2018) details social and structural considerations of PrEP uptake across a PrEP continuum of care, some of which overlaps with the findings of this manuscript.*

**Reply 9:** We are grateful for this comment, which has been fundamental to this major revision of our manuscript. In the new paragraph on the PrEP continuum, we no longer focus only on the model developed by Nunn et al. (2017) but make reference to all of the models we could identify in the literature and through the references suggested by the reviewer. Additionally, we describe the distinction between (a) the models that reflect a more linear, biomedical approach and (b) the augmented PrEP cascade of Newman et al. (2018). We also make the point that PrEP is not a desired or viable option for all MSM even more clearly than we had previously, and we take care not to describe the continuum as if it were linear (e.g., we changed our phrase ‘one step to the next’ to ‘one step to another’, l. 148). Lastly, we have added a phrase at the end of the paragraph that emphasises the importance of the broader sexual and psychological health of MSM. The paragraph now reads as follows:

‘To improve the implementation of PrEP, a range of authors over the past decade have proposed and refined several models of a continuum or “cascade” of PrEP care [31-37]. Most of the earlier models are largely based on a biomedical approach that assumes HIV prevention is the main priority in a person’s sexual decision-making and therefore evaluate this decision-making chiefly in terms of risk [38] and in a linear fashion. For example, the model proposed by Nunn et al. (2017) comprises nine steps ranging from identifying individuals at the highest risk of HIV to an end goal of retaining individuals in PrEP care [33]. In contrast, one of the more recent iterations of the PrEP care continuum, developed by Newmann et al. (2018), follows a more holistic notion of personhood rooted in the results of their own qualitative analysis and the extensive anthropological and ethnographic literature on HIV prevention [37]. Their model considers the psychological, interpersonal, social and community phenomena that affect PrEP use and non-use, resulting in an augmented PrEP cascade that incorporates alternative decision-making, pathways (such as those with the end goal of intermittent use or discontinuation), and psychosocial impacts [37]. Regardless of the epistemological roots and approaches of these different models, however, what all of them share is that each stage in the proposed cascade represents a potential point of intervention to improve PrEP implementation among those for whom PrEP is a desired and viable option [32, 33]. At the same time, each of these steps is characterised by unique but overlapping sets of barriers and enablers that must be considered if we are to understand more fully how and why individuals move from one step to another – an understanding that is crucial to developing interventions to improve the implementation of PrEP, and HIV prevention strategies more generally, that are efficient, effective and, ideally, support the broader sexual and psychological health of MSM.’ (ll. 121-151)

***Comment 10:*** *Moreover, given the topic of the paper is on motivations of PrEP use, I believe that the sentence “However, our knowledge about explicit motivations that influence decisions to initiate PrEP is less well developed and stems mostly from small qualitative studies involving focus groups or interviews [38-42]” warrants its own paragraph and more details. There is also additional research on motivations for PrEP use, some of which corroborates the findings of the researchers, that could be integrated into the introduction and the discussion, for example:*

[*https://sti.bmj.com/content/95/4/262.abstract*](https://sti.bmj.com/content/95/4/262.abstract)

*https://academic.oup.com/abm/article/49/2/177/4561523?login=true*

*Then, it may be helpful to add one additional statement explicitly summarizing gaps in the evidence base.*

**Reply 10:** We agree with this very helpful comment in full and acknowledge that we had given short shrift to the previous literature on this point, also in terms of how we originally framed the rationale for our study. Based on this comment and a similar one made by Reviewer #2, we have completely revised this section of the manuscript, as follows:

1) In the paragraph following the PrEP continuum paragraph, we still start by outlining barriers identified in previous literature, but we have expanded the breadth of references and reordered the categories of barriers to better reflect their reporting in the literature. We then end the paragraph with a statement that acknowledges the previous work on motivations of PrEP use, as follows:

‘Until recently, one significant gap in the literature was the lack of data on individuals’ motivations for taking PrEP. Indeed, in their comprehensive early review of research on the acceptability of PrEP and treatment as prevention (TasP), Young and McDaid (2014) identified motivations for PrEP use and adherence as one of the main areas in which further research was needed in order to identify “when, where and for whom PrEP and TasP would be most appropriate and effective” [45]’. (ll. 153-170)

2) We then begin a new paragraph in which we go into greater detail and provide a rough overview of the motivations identified in the qualitative literature while leaving a more in-depth discussion of them and their importance (both in general and with regard to our findings) to the Discussion section. The new paragraph in the Introduction begins as follows:

‘A number of researchers, particularly in anthropological, ethnographic and other primarily qualitative lines of enquiry, have since stepped up to the plate to examine this topic. By exploring the subjective experiences of MSM in their navigation of PrEP, these authors have identified, mostly through in-depth interviews or in focus groups, numerous and varied motivations for using (or not using) PrEP that call into question the usefulness of biomedical approaches to PrEP implementation that de-emphasise psychosocial phenomena. In many cases, the motivations identified in these studies have stemmed from powerful affective experiences, such as’ (examples of specific motivations and references to the studies follow this) (entire paragraph is from ll. 172-189)

3) This is followed by a paragraph in which we give some examples of the quantitative research undertaken on this topic to date, but in which we also point out that ‘Among the studies that have explored motivations using survey-based quantitative methods, many have approached the subject in an indirect fashion using, for instance, correlation or regression analysis to identify factors associated with PrEP use, non-use, willingness to use PrEP or PrEP acceptability.’ (ll. 191-194). Later in the paragraph we explain more clearly the rationale for our study:

‘Indeed, among this latter stream of the quantitative literature, we were unable to identify any surveys that explicitly asked MSM to name or describe their motivations for taking PrEP, whether by providing them with a list of pre-specified motivations or the possibility of writing a free-text response.’ (see entire paragraph at ll. 191-210)

4) Subsequently, we add to this re-framing of the rationale for our study by pointing out some potential methodological advantages of our approach (as an addition to existing qualitative studies) and also highlight that much of the literature on this subject is restricted to the US context (as well as Australia, the United Kingdom and Canada). (see entire paragraph at ll. 212-233) Please note that we have also reframed the rationale for the study accordingly in the Background section of the Abstract, as follows:

‘While our knowledge of what motivates men who have sex with men (MSM) to use HIV pre-exposure prophylaxis (PrEP) has grown in recent years, quantitative survey-based studies have not asked MSM explicitly to name their motivations. We did so using a qualitative open-ended question and aimed to categorise the responses and explore whether these were related to where MSM were located along a conceptual continuum of PrEP care.’ (ll. 42-47)

5) Lastly, in the Discussion, we revisit the qualitative literature on this subject, pointing out why it is important and reflecting on how our results fit into it, as follows (please also see our separate paragraph on the literature on an holistic approach, which we also describe below in this point-by-point reply):

‘This latter finding resonates with the results of qualitative studies that have engaged with this area of enquiry, challenging the predominant biomedical approach to PrEP implementation. With regard to motivations such as those to reduce HIV anxiety, improve self-efficacy, be able to make one’s own informed choices, and feel less fear and shame, for example, our respondents wrote short but powerful responses about “not constantly having to be afraid,” living a “whole life” in which “there’s only been sex with a condom and fear of HIV”, wanting to “try a few sex partners who I otherwise couldn’t without being on PrEP”, and no longer having to “feel regret” or have “psycho-stress” after having “unprotected” or “unsafe” sex. Examining such phenomena, and affective experiences more generally, is crucial because these have been shown to have a profound impact on the sexual and psychological well-being of MSM (e.g., [35, 37, 38, 57, 59, 60]). Moreover, these motivations are also tied to ongoing decision-making about PrEP that is based on sexual risk and relationship trajectories that have been shown to be varied and dynamic [37].’ (ll. 603-616)

***Comment 11:*** *Minor notes: line 78 at first mention of United States include US in brackets, as you use US throughout after that; also, MSM should be spelled out at first mention.*

**Reply 11:** Done in both cases (l. 84 and l. 83, respectively).

***MATERIALS AND METHODS***

***Comment 12:*** *I appreciated the thorough overview of recruitment, and in particular the in-depth description of the non-municipal counselling and testing centers and HIV specialist services.*

**Reply 12:** Thank you. We have now added to this description a statement that non-municipal counselling and testing centres offer low-barrier services, and that the target audience includes adolescents. We have also added that the HIV specialist practices also take walk-ins. (Changes made in ll. 267-275)

***Comment 13:*** *Was there a rationale for randomly selecting 30 out of 228 responses to create the coding framework? For example, did the authors reach some level of saturation (no new category generation) by open-text response 30? Upon coding the remaining free-text responses, were there any motivations that did not fit within the originally-developed framework? If so, how did the authors resolve this?*

**Reply 13:** This comment is important because it makes clear to us that while we provided a detailed description of the deductive side of our approach to coding, our description of the inductive side was inadequate. Also, the comment highlights a typographic error in our paper: the pilot coding involved 75 open-text responses, not 30. Of course, 75 is also an arbitrary number, so we have added an explanation for it. In total, to address these concerns, we have now made the following changes:

1) In addition to various minor edits and clarifications, we added a sentence that explained why we took a combined deductive and inductive approach to coding, as follows: ‘To help ensure that the analysis was not driven primarily by our theoretical interests and analytic preconceptions, we took a combined deductive and inductive approach to developing the coding framework [66, 67]’ (Change in ll. 327-329)
2) We added the following paragraph to the section now called ‘Qualitative data analysis’:

‘The second researcher (MS), who developed a set of categories and subcategories inductively, took the following approach: first, the third researcher (MG) familiarised himself with the entire qualitative dataset by reading and re-reading all free-text response to make a pragmatic assessment of the number of these that would need to be coded before saturation was reached (i.e., roughly one third of all valid responses). Subsequently, a list of randomly generated numbers was appended to the qualitative dataset, and the responses numbered between one and 75 were sent to the second researcher for pilot coding. Coding was undertaken in a manner that was both open (i.e., with codes being assigned to describe as many perspectives as possible, such as particular behaviours, intentions, values, beliefs and emotions) [66] and circular (i.e. looping or cycling repeatedly between the qualitative codes assigned to the data and the data itself, and recoding, considering emergent categories, and recategorising as needed) [72].’ (ll. 355-367)

3) We added a paragraph to explain in greater detail the approach taken to coding the remaining free text responses. We are conscious that the paper is quite long, and if the reviewer feels we have gone into too much detail here, we are happy to shorten the paragraph. The new paragraph reads as follows:

‘Two researchers (RNW, MS) subsequently used this framework independently to identify, code and classify individual motivations in each free-text response (including the 75 responses used in the pilot coding). Meetings among all three researchers were held at regular intervals during the coding and classification process to discuss potential emerging new categories or subcategories during the coding. Ultimately, no substantial changes to the framework were needed during this process, although we did clarify the definition for the subcategory “Expectations of worry free-sex” to require, in contrast to the subcategory “Reducing anxiety, fear, or worries of being infected with HIV”, the explicit mention of sex or sexuality. Moreover, a subcategory “Desire to increase quality of life or sexual freedom” was broadened to include “sexual/personal freedom”. Furthermore, even though the number of responses in the category “Norms / social perspectives” was minimal, we decided to retain it because of its prominence as a finding in the qualitative literature (e.g., [37, 58, 61]) and our desire not to take an overly frequentist approach to reporting the qualitative data. Lastly, while preparing the manuscript we decided to use the term “quality of life” instead of “general health” to describe the category “Mental health and quality of life” because we felt the term better captured the nature of the responses we had assigned to various subgroups in this category.’ (ll. 381-399)

***Comment 14:*** *In terms of structure, I believe it would be clearer if the sub-heading ‘Literature search’ was removed and the literature search paragraph integrated into the analysis section before the line “These two classifications systems…”.*

**Reply 14:** We agree and have implemented this change as suggested. The change also makes it clearer that we used a combined deductive and an inductive approach to developing the coding framework. (A comment by Reviewer 2 suggested to us that we had not been clear enough in this regard.) (Changes made in ll. 339-353 and ll. 406-423)

***Comment 15:*** *Moreover, the operationalization of the PrEP care continuum is related to the quantitative analysis and could be absolved into that section before the line beginning with “For any post-hoc pairwise comparisons…”*

**Reply 15:** We have implemented this as suggested and feel it makes the multi-methods nature of our approach much clearer. Thank you. (Please note that we have removed the detailed description of Nunn et al.’s model for a somewhat more general description of three models (Newmann et al., Nunn et al., and Parsons et al.). We felt that there was no need to highlight any particular model in this paragraph given that the distinction between our two groups (i.e., never users vs. current or history of PrEP use) is not as fine-grained as the distinction between individual stages in any of these models. (See paragraph in ll. 436-462)

***Comment 16:*** *Table 1: I appreciate the authors’ integration of references. Some of the suggested references above may also be able to be integrated into the table so that there are references for most of the sub-categories (E.g., Newman et al. (2018) could be used to reference ‘expectations of more pleasureful sex or increased intimacy and closeness (when not using a condom)’, ‘ expectations of worry-free sex’, ‘desire to increase quality of life or sexual/personal freedom’, ‘reducing anxiety, fear, or worries of being infected with HIV’, and ‘autonomy and self-empowerment in the protection against HIV’, among other sub-categories.*

**Reply 16:** Thank you for this suggestion. We have integrated into the table the references suggested by the reviewer, as well as other references we identified as part of our revision to better recognise and reflect the large amount of previous work that has been undertaken in this area. (Multiple changes made to Table 1, pp. 16-17)

***Comment 17:*** *One aspect I wondered about in the Abstract, and now here, is the potential overlap between ‘worry-free sex’ and ‘reducing anxiety, fear, or worries of being infected with HIV’ – can the authors expand more on how they are conceptualizing the difference between these sub-categories throughout the manuscript.*

**Reply 17:** Thank you for pointing out this potential source of misunderstanding. In the latter subcategory we had already mentioned that ‘Unlike the subcategory ‘expectations of worry-free sex’, this subcategory does not include responses that refer explicitly to the act of sex.’. However, we have now added further clarification to Table 1 and in the abstract as follows:

1) In Table 1: ‘In contrast to the subcategory “Reducing anxiety, fear, or worries of being infected with HIV” under the category “Mental well-being and quality of life”, responses here had to mention sex or sexuality explicitly.’ (p. 16)
2) Abstract: “…(4) Expectations about sexuality (14.4%, including worry-free sex or more pleasurable sex, with explicit mention of sex or sexuality)” (l. 66, underline added in this point-by-point reply to highlight change, but not in manuscript itself)
3) In the Methods section under the new heading ‘Qualitative data analysis’ we have included the following sentence (among others describing how we dealt with emerging categories, etc., during the coding process): ‘Ultimately, no substantial changes to the framework were needed during this process, although we did clarify the definition for the subcategory “Expectations of worry free-sex” to require, in contrast to the subcategory “Reducing anxiety, fear, or worries of being infected with HIV”, the explicit mention of sex or sexuality.’ (see entire paragraph at ll. 381-399)

***Comment 18****: For clarity, I suggest using the sub-headings ‘Qualitative data analysis’ (for section that is now called Data analysis) and then ‘Quantitative data analysis’ (for the section that is now called Sample size and statistical methods).*

**Reply 18:** We agree and feel that the new headings make both our approach and the structure of our paper clearer. Thank you. (Changes made in l. 323 and l. 426)

***Comment 19****: The way that the study is currently depicted is as a multi-method as opposed to a mixed-methods study. Mixed methods is an in-depth methodology that goes beyond the rigorous collection and analysis of both quantitative and qualitative data to include the integration of the two forms of data using a pre-specified mixed methods typology (See: Creswell & Plano-Clark, 2017), thus, more details about the mixed-methods methodology should be included. As the key to mixed-methods is integration of quantitative and qualitative data, if mixed-methods is maintained as the methodology, another section should be added titled ‘Mixed methods analysis’ that details how quantitative and qualitative data were compared/contrasted (See: Fetters, Curry, & Creswell, 2013). Alternately, the authors may opt to refer to their study as a multi-method study.*

**Reply 19:** Thank you for this. We have reviewed the references suggested by the reviewer and agree that our study methodology cannot be described as ‘mixed methods’ according to the criteria therein. We have now opted, as the reviewer suggests, to refer to our study as a ‘multi-method study’. (i.e. ‘mixed’ replaced with ‘multi’ throughout manuscript)

***Comment 20:*** *Minor: Please spell in full at first use of LGBTI+.*

**Reply 20:** Done. We now write ‘…the sexual health of lesbian, gay, bisexual, transgender, intersex and other gender diverse (LGBTI+) people…’ when the abbreviation is first used (ll. 270-271).

***RESULTS***

***Comment 21:*** *The first paragraph of the results could be integrated within the methods section ‘Sampling methods, participants, and settings’, up until the last line; the last line could be included in the section ‘Demographic data, sexual behavior, and HIV risk of analysis sample’.*

**Reply 21:** We have implemented this suggestion in full. (See ll. 300-312 and ll. 492-494)

***Comment 22:*** *Table 2 – rows with 0 responses (e.g., primary education, small town/rural area in Germany) could be omitted.*

**Reply 22:** While we agree that deleting these lines in the table would make it more compact and therefore easier to read, we feel that it is important to include these items so that readers who are not inclined to read through our survey questionnaire will know that these answers were available as options to the participants. If the reviewer does not object, we would prefer to retain these two lines.

***Comment 23:*** *The lines “There was a high degree of agreement between the coding results…” should be integrated within the qualitative data analysis sub-section. The subsection coding of responses could be more appropriately titled Motivations of Participants (or something similar). I don’t think the line “there were no disagreements between the reviewers in this regard” is needed – if maintained, it should also be moved to the analysis.*

**Reply 23:** We agree with all of these suggestions and have implemented them in full as suggested. (see ll. 401-402 and l. 516)

***Comment 24:*** *I may have missed it, but I’m not clear how the authors classified a motivation as a main motivation – does this just mean that the participant wrote it down as a motivation, or did the authors denote some order as to what was main when participants indicated more than one motivation? Please clarify.*

**Reply 24:** Thank you for pointing out our confusing wording here. We asked participants about their main motivation (in German: Hauptmotivation) for taking or considering HIV PrEP, and that is all we were referring to with the term ‘main motivation’. You are right: We did not denote an order when participants indicated more than one motivation because we did not feel that simply listing one motivation before another meant that it could be classified as more important. To address this, we have changed the term ‘main motivation’ to ‘motivation’ throughout the manuscript, unless of course we are referring specifically to the question on the questionnaire itself. (i.e., ‘main’ deleted throughout the manuscript except when referring specifically to the survey item)

***DISCUSSION***

***Comment 25:*** *I am wondering if the authors have more context about provider concern about condom use as leading to hesitancy about PrEP – is this an issue in the EU context that has prevented widespread adoption of PrEP into public insurance plans? Additional references would be helpful.*

**Reply 25:** This is a good point. First, we have changed our description from ‘widespread abandonment of condom use’ to ‘sexual risk compensation’ (ll. 589-591); secondly, we indeed could not identify recent data on such a hesitancy (beyond anecdotal reports), so we have deleted the word ‘still’ in the original sentence. We now write the following, which we hope is more circumspect, and include two references from the European context:

‘Even though some 18% of respondents in our analysis sample explicitly mentioned a desire or intent to engage in condomless sex as a motivation for using or considering PrEP, these findings about safety and protection should reassure payers and practitioners who might be focusing disproportionately on the subject of sexual risk compensation, i.e., an increase in sexual behaviours that could put those who engage in them at a higher risk of infection with HIV and other STIs [75-80].’ (ll. 586-593)

***Comment 26:*** *The authors may also consider a broader discussion of literature pertaining to PrEP counselling from a holistic lens, as they are describing, see:*

*https://academic.oup.com/hsw/article-abstract/46/1/22/6151208*

*https://link.springer.com/article/10.1007/s10461-020-02855-9*

*https://www.tandfonline.com/doi/abs/10.1080/09540121.2020.1744507*

**Reply 26:** This comment, alongside the reviewer’s earlier comments about the PrEP continuum paragraph in our Introduction section, has helped us to draw out the implications of our findings with regard to broader mental and sexual health issues, and therefore improve our paper substantially. Thank you for this. In the Discussion section, we have implemented this specific suggestion by adding the following paragraph, which incorporates the literature suggested by the reviewer and reflects on how it relates to our findings:

‘The idea of taking a more holistic approach to PrEP implementation has been the focus of several recent publications. Lacombe-Duncan et al. (2021) drew on qualitative data from in-depth, semi-structured interviews with 29 MSM recruited purposively in 2015/16 in a community setting in Toronto, Canada, to identify and explore gaps in PrEP implementation that might be amenable to social work intervention [61]. Among other results, they found that MSMs’ navigation of sexual health and risk practices was made complex by a range of sometimes contradictory factors encompassing the individual, interpersonal, organisational and structural levels. This aligns very much with the breadth of motivations reported in our survey, as well as the reporting of multiple motivations by individual respondents. Lacombe-Duncan et a. (2021) make clear that addressing such factors and, more specifically the social determinants of health and psychosocial issues that affect PrEP engagement, solely in terms of HIV infection risk and a binary decision about PrEP uptake would be wholly inadequate to support individuals with informed decision making [61]. A recently published systematic review by Pinto et al. (2018) corroborates these and, in part, our findings, and identifies the need for a range of multilevel interventions targeting multiple socioecological domains, such as interventions to help people navigate health care systems and improve referrals to mental health and supportive services, as well as provider-level interventions such as increased training and education about PrEP [43]. In their scoping review of PrEP service delivery and programming, Hillis et al. (2020) also identify a range of potentially useful multidisciplinary and innovative PrEP care pathways; however, they make the important additional point that PrEP provision in a health system actually creates new opportunities for MSM to access health services to which they might not otherwise have availed themselves, such as sexual health care, testing, treatment, counselling and STI testing and psychological support [87].’ (ll. 645-669)

In the paragraph preceding this, we also added the term ‘social worker’ to the list of health professionals in the following sentence: ‘While this may be something that any good doctor, or sexual health counsellor or social worker would do, the results of our study might nevertheless be useful as a reminder of the breadth of motivations that can influence the decision to take use PrEP and encourage practitioners to seek to identify multiple (and, indeed, even apparently contradictory) factors in individual patients.’ (ll. 636-640)

***Comment 27:*** *Excellent overview of the limitations of the study.*

**Reply 27:** Thank you.

**RESPONSES TO REVIEWER #2:**

***Comment 1:*** *This paper reviews the motivations for PrEP use among a sample of MSM living in Berlin. It is methodologically sound and well-written with some thought-provoking conclusions, particularly with respect to the mental health circumstances of MSM and how these may be alleviated, for some, by the use PrEP. There are, however, a few issues that I think need to be addressed before it is ready for publication.*

**Reply 1:** Thank you very much for your detailed and helpful comments. We have implemented them as outlined in our replies below and feel that our manuscript has benefitted greatly as a result.

***Comment 2:*** *Recognising and reflecting previous research. The emergence of PrEP has seen a significant number of papers published that examine: the acceptability of PrEP; motivations for PrEP use; factors that may influence PrEP uptake and continued use; how PrEP is integrated into sexual lives; how PrEP reforms how MSM think about HIV risk, etc. As the key rationale for the study, the authors states that most prior research has been focus group or interview based and, therefore, larger scale data is required. I’m not sure this accurately reflects the nature of the literature on this topic (where there are a number of quantitative or survey-based studies) nor does being ‘bigger’ necessarily constitute a clear rationale for this study. I would encourage the authors to acknowledge the large amount of quantitative literature on this topic (look, for example, at work by Bavinton, Prestage and Holt, among others) and consider recasting the principle rationale for the study to identify what is unique.*

**Reply 2:** Thank you very much for this comment, which echoes several comments and suggestions made by Reviewer #1. We have now thoroughly revised the part of the Introduction section that outlines the previous literature, and in the revised Discussion section we now strive to recognise and reflect on previous research as well. These revised sections are too long to include in here in the point-by-point reply, so we summarise them below with line number references indicating the full paragraphs and would kindly ask the reviewer to refer to these:

1) In the paragraph following the PrEP continuum paragraph, we still start by outlining barriers identified in previous literature, but we have expanded the breadth of references and reordered the categories of barriers to better reflect their reporting in the literature. We then end the paragraph with a statement that acknowledges the previous work on motivations of PrEP use, as follows:

‘Until recently, one significant gap in the literature was the lack of data on individuals’ motivations for taking PrEP. Indeed, in their comprehensive early review of research on the acceptability of PrEP and treatment as prevention (TasP), Young and McDaid (2014) identified motivations for PrEP use and adherence as one of the main areas in which further research was needed in order to identify “when, where and for whom PrEP and TasP would be most appropriate and effective” [45]’. (ll. 153-170)

2) We then begin a new paragraph in which we go into greater detail and provide a rough overview of the motivations identified in the qualitative literature while leaving a more in-depth discussion of them and their importance (both in general and with regard to our findings) to the Discussion section. The new paragraph in the Introduction begins as follows:

‘A number of researchers, particularly in anthropological, ethnographic and other primarily qualitative lines of enquiry, have since stepped up to the plate to examine this topic. By exploring the subjective experiences of MSM in their navigation of PrEP, these authors have identified, mostly through in-depth interviews or in focus groups, numerous and varied motivations for using (or not using) PrEP that call into question the usefulness of biomedical approaches to PrEP implementation that de-emphasise psychosocial phenomena. In many cases, the motivations identified in these studies have stemmed from powerful affective experiences, such as’ (examples of specific motivations and references to the studies follow this) (entire paragraph is from ll. 172-189)

3) This is followed by a paragraph in which we give some examples of the quantitative research undertaken on this topic to date, but in which we also point out that ‘Among the studies that have explored motivations using survey-based quantitative methods, many have approached the subject in an indirect fashion using, for instance, correlation or regression analysis to identify factors associated with PrEP use, non-use, willingness to use PrEP or PrEP acceptability.’ (ll. 191-194). Later in the paragraph we explain more clearly the rationale for our study:

‘Indeed, among this latter stream of the quantitative literature, we were unable to identify any surveys that explicitly asked MSM to name or describe their motivations for taking PrEP, whether by providing them with a list of pre-specified motivations or the possibility of writing a free-text response.’ (see entire paragraph at ll. 191-210)

4) Subsequently, we add to this re-framing of the rationale for our study by pointing out some potential methodological advantages of our approach (as an addition to existing qualitative studies) and also highlight that much of the literature on this subject is restricted to the US context (as well as Australia, the United Kingdom and Canada). (see entire paragraph at ll. 212-233) Please note that we have also reframed the rationale for the study accordingly in the Background section of the Abstract, as follows:

‘While our knowledge of what motivates men who have sex with men (MSM) to use HIV pre-exposure prophylaxis (PrEP) has grown in recent years, quantitative survey-based studies have not asked MSM explicitly to name their motivations. We did so using a qualitative open-ended question and aimed to categorise the responses and explore whether these were related to where MSM were located along a conceptual continuum of PrEP care.’ (ll. 42-47)

5) Lastly, in the Discussion, we revisit the qualitative literature on this subject, pointing out why it is important and reflecting on how our results fit into it, as follows (please also see our separate paragraph on the literature on an holistic approach, which we also describe below in this point-by-point reply):

‘This latter finding resonates with the results of qualitative studies that have engaged with this area of enquiry, challenging the predominant biomedical approach to PrEP implementation. With regard to motivations such as those to reduce HIV anxiety, improve self-efficacy, be able to make one’s own informed choices, and feel less fear and shame, for example, our respondents wrote short but powerful responses about “not constantly having to be afraid,” living a “whole life” in which “there’s only been sex with a condom and fear of HIV”, wanting to “try a few sex partners who I otherwise couldn’t without being on PrEP”, and no longer having to “feel regret” or have “psycho-stress” after having “unprotected” or “unsafe” sex. Examining such phenomena, and affective experiences more generally, is crucial because these have been shown to have a profound impact on the sexual and psychological well-being of MSM (e.g., [35, 37, 38, 57, 59, 60]). Moreover, these motivations are also tied to ongoing decision-making about PrEP that is based on sexual risk and relationship trajectories that have been shown to be varied and dynamic [37].’ (ll. 603-616)

***Comment 3:*** *Acceptable in theory and in practice. Partially linked to the point above, prior to the widespread rollout of PrEP there were a large number of studies looking at the theoretical acceptability of PrEP (see review by Ingrid Young). What I found myself wondering was how the findings of your study – when people CAN actually access and use PrEP differ from/relate to the motivations MSM provided when talking about PrEP in the abstract. Can you reflect some of this literature in your discussion?*

**Reply 3:** This is an important question. In the review by Young et al. (2013), the authors report on measures of acceptability and willingness (as a proxy for acceptability) in the studies included in their review, but they go on to point out that studies on motivations are lacking. We now include this observation in our revised introduction and then give an overview of some of the relevant qualitative and quantitative work in this area, as outlined in Reply 2 above.

This being said, Young et al. (2013), among others, do indeed report on some psychosocial correlates of a lack of willingness to use PrEP, which one could interpret as barriers/motivations not to take PrEP. In the paragraph in which we describe the results of our analysis of whether motivations differed between participants at two different locations on a conceptual continuum of PrEP care, we have now added the following sentences to explore/hypothesise about the before and after question the review has posed.

‘Interestingly, the comprehensive review of the literature undertaken by Young et al. (2013) before the wide rollout of PrEP found that willingness to take PrEP (the closest construct to motivations that they examined) was associated with younger age, unprotected anal intercourse with casual partners and increased risk perceptions; on the other side, anxieties around medical side effects of PrEP were commonly reported in the included studies as barriers. Psychosocial correlates of willingness, such as reduced anxiety about HIV infection or an increase in self-efficacy, do not appear to have been reported as frequently in this pre-rollout phase, however. While this may simply be an artefact of the biomedical approach to these questions taken by many of the studies included in their review, it might also support the hypothesis that these positive aspects of PrEP only become more readily apparent after PrEP use has been initiated.’ (ll. 687-698)

***Comment 4:*** *Coding frame. Can you provide a clear rationale as to why you created a coding frame from a review of the literature and applied it to your data, rather than working with the data in an inductive manner? I also think it would be much easier to follow if you describe the literature review process, and its purpose, prior to presenting the coding frame.*

**Reply 4:** Thank you very much for this comment. To create a coding frame, we used a combined deductive and inductive approach: one researcher created a frame deductively based on a review of the literature and another researcher created a frame by pilot coding a sample of the data in an inductive manner. The two frames were then merged by the two researchers through discussion, with discrepancies and disagreements resolved by consensus in meetings facilitated by a third researcher. This merged frame was then used to code all responses (including that had been pilot coded). However, based on your helpful comment and a related comment from Reviewer #1, we now realise that we did not describe this approach clearly enough. In particular, as you point out, having a separate section in the paper to describe the literature review process created confusion. To address this and to clarify our approach, we have made the following changes:

1) In the data analysis section (now called ‘Qualitative data analysis’), we have added the following phrase to the sentence in which we introduce our approach: ‘To help ensure that the analysis was not driven primarily by our theoretical interests and analytic preconceptions, we took a combined deductive and inductive approach to developing the coding framework [66, 67]’ (ll. 327-329).
2) We have removed the sub-heading ‘Literature search’ and have now integrated the literature search paragraph into the ‘Qualitative data analysis’ section before the line ‘These two classifications systems…’ (ll. 334-353) (This was also suggested by Reviewer #1.)
3) We have added a paragraph describing in greater detail the inductive approach taken by the second researcher. (Suggested by Reviewer #1.) This paragraph reads as follows:

‘The second researcher (MS), who developed a set of categories and subcategories inductively, took the following approach: first, the third researcher (MG) familiarised himself with the entire qualitative dataset by reading and re-reading all free-text response to make a pragmatic assessment of the number of these that would need to be coded before saturation was reached (i.e., roughly one third of all valid responses). Subsequently, a list of randomly generated numbers was appended to the qualitative dataset, and the responses numbered between one and 75 were sent to the second researcher for pilot coding. Coding was undertaken in a manner that was both open (i.e., with codes being assigned to describe as many perspectives as possible, such as particular behaviours, intentions, values, beliefs and emotions) [66] and circular (i.e. looping or cycling repeatedly between the qualitative codes assigned to the data and the data itself, and recoding, considering emergent categories, and recategorising as needed) [72].’ (ll. 355-367)

4) We now describe the approach in the Methods part of the Abstract as follows: ‘One researcher developed a thematic framework deductively from the literature and another did so inductively from the free-text data, and a merged framework was used to code responses independently.’ (ll. 52-54)

***Comment 5:*** *As a minor point, can you include a short summary as to the status of PrEP availability in Germany at the time of data collection? Where could it be obtained? How much did it cost? What prescribing criteria has to be met? Such information would aid interpretation from an international audience.*

**Reply 5:** Thank you. We are happy to provide this additional context for international readers and have added the paragraph below to the section ‘Sampling methods, participants and settings’. However, we are also conscious that our paper is long, and if the reviewers feel it needs to be shortened, this might be one paragraph that could be deleted given that our open-ended question was not designed to elicit information on barriers such as cost.

‘During the data collection period (1 October 2017 to 2 April 2018), PrEP was available in Germany only by means of a private prescription, which could be written by any licensed doctor. About half way through this period, the price of a month’s supply of generic PrEP in Germany fell from approximately 600 euros to as low as 50 euros [30]. The relevant guideline for identifying at-risk patients and prescribing PrEP is the German and Austrian Guideline on HIV pre-exposure prophylaxis [65], but its recommendations are not binding. The German system of statutory health insurance began to cover the costs associated with PrEP in September 2019.’ (ll. 291-298)

**FOR ACADEMIC EDITOR/BOTH REVIEWERS**

**MINOR EDITORIAL CHANGES:**

- The phrase in the Introduction ‘facility-based survey of PrEP use among MSM in Berlin in 2017/18, of which the current study is a part: in the survey, 12.9% of participants who reported never having used PrEP strongly agreed with the statement that they would like to do so’ was changed to ‘facility-based paper survey of PrEP use among MSM (N=470) in Berlin in 2017/18, of which the current study is a part: in the survey, 12.9% of participants who reported never having used PrEP (who comprised 82.3% of the sample) strongly agreed with the statement that they would like to do so’ (ll. 111-116)
- ‘Self-assigned qualification’ (translation error from the German) was changed to ‘self-perceived eligibility’ (Table 1, p. 17)
- We changed ‘spread’ to ‘geographic spread’ in the following sentence in the Methods section ‘We chose these purposively based on their spread across Berlin’ (l. 286)
- We changed ‘motivations for hesitating to use or abstaining from using PrEP’ to ‘motivations for not using PrEP’ in the limitations section (l. 769)
- We changed the translation of ‘weniger Kopfkino’ from ‘Calming down my mental movie projector’ to ‘Less chance for my imagination to run away from me’ (Table 4, p. 29)
